# Expanding Clinical Presentations Due to Variations in THOC2 mRNA Nuclear Export Factor

**DOI:** 10.3389/fnmol.2020.00012

**Published:** 2020-02-11

**Authors:** Raman Kumar, Elizabeth Palmer, Alison E. Gardner, Renee Carroll, Siddharth Banka, Ola Abdelhadi, Dian Donnai, Ype Elgersma, Cynthia J. Curry, Alice Gardham, Mohnish Suri, Rishikesh Malla, Lauren Ilana Brady, Mark Tarnopolsky, Dimitar N. Azmanov, Vanessa Atkinson, Michael Black, Gareth Baynam, Lauren Dreyer, Robin Z. Hayeems, Christian R. Marshall, Gregory Costain, Marja W. Wessels, Julia Baptista, James Drummond, Melanie Leffler, Michael Field, Jozef Gecz

**Affiliations:** ^1^Adelaide Medical School and the Robinson Research Institute, The University of Adelaide, Adelaide, SA, Australia; ^2^Genetics of Learning Disability Service, Hunter Genetics, Waratah, NSW, Australia; ^3^School of Women’s and Children’s Health, University of New South Wales, Randwick, NSW, Australia; ^4^Faculty of Biology, Medicine and Health, Division of Evolution and Genomic Sciences, School of Biological Sciences, University of Manchester, Manchester, United Kingdom; ^5^Manchester Centre for Genomic Medicine, St. Mary’s Hospital, Manchester University NHS Foundation Trust, Health Innovation Manchester, Manchester, United Kingdom; ^6^Department of Neuroscience, Erasmus MC University Medical Center, Rotterdam, Netherlands; ^7^ENCORE Expertise Centre for Neurodevelopmental Disorders, Erasmus MC University Medical Center, Rotterdam, Netherlands; ^8^Genetic Medicine, Department of Pediatrics, University of California, San Francisco, San Francisco, CA, United States; ^9^North West Thames Regional Genetics Service, Northwick Park Hospital, Harrow, United Kingdom; ^10^Nottingham Clinical Genetics Service, Nottingham University Hospitals NHS Trust, and the 100,000 Genomes Project and the Genomics England Research Consortium, Nottingham, United Kingdom; ^11^Division of Pediatric Neurology, Medical University of South Carolina, Charleston, SC, United States; ^12^Department of Pediatrics, McMaster University Medical Centre, Hamilton, ON, Canada; ^13^Department of Diagnostic Genomics, PathWest, Nedlands, WA, Australia; ^14^Division of Pathology and Laboratory Medicine, Medical School, University of Western Australia, Crawley, WA, Australia; ^15^Faculty of Health and Medical Sciences, University of Western Australia Medical School, Perth, WA, Australia; ^16^Genetic Services of Western Australia, Undiagnosed Diseases Program, Department of Health, Government of Western Australia, Perth, WA, Australia; ^17^Linear Clinical Research, Perth, WA, Australia; ^18^Child Health Evaluative Sciences, Research Institute, The Hospital for Sick Children, and Institute of Health Policy Management and Evaluation, University of Toronto, Toronto, ON, Canada; ^19^Genome Diagnostics, Department of Paediatric Laboratory Medicine, The Hospital for Sick Children, and Laboratory Medicine and Pathobiology, University of Toronto, Toronto, ON, Canada; ^20^Department of Paediatrics, Division of Clinical and Metabolic Genetics, The Hospital for Sick Children, Toronto, ON, Canada; ^21^Department of Clinical Genetics, Erasmus MC University Medical Center, Rotterdam, Netherlands; ^22^Royal Devon and Exeter NHS Foundation Trust, Exeter, United Kingdom; ^23^Neuroradiology, Royal North Shore Hospital, Sydney, NSW, Australia; ^24^Childhood Disability Prevention, South Australian Health and Medical Research Institute, Adelaide, SA, Australia

**Keywords:** mRNA export, THOC2, intellectual disability, neurodevelopmental disorders, microdeletion

## Abstract

Multiple TREX mRNA export complex subunits (e.g., THOC1, THOC2, THOC5, THOC6, THOC7) have now been implicated in neurodevelopmental disorders (NDDs), neurodegeneration and cancer. We previously implicated missense and splicing-defective *THOC2* variants in NDDs and a broad range of other clinical features. Here we report 10 individuals from nine families with rare missense *THOC2* variants including the first case of a recurrent variant (p.Arg77Cys), and an additional individual with an intragenic *THOC2* microdeletion (Del-Ex37-38). *Ex vivo* missense variant testing and patient-derived cell line data from current and published studies show 9 of the 14 missense THOC2 variants result in reduced protein stability. The splicing-defective and deletion variants result in a loss of small regions of the C-terminal THOC2 RNA binding domain (RBD). Interestingly, reduced stability of THOC2 variant proteins has a flow-on effect on the stability of the multi-protein TREX complex; specifically on the other NDD-associated THOC subunits. Our current, expanded cohort refines the core phenotype of THOC2 NDDs to language disorder and/or ID, with a variable severity, and disorders of growth. A subset of affected individuals’ has severe-profound ID, persistent hypotonia and respiratory abnormalities. Further investigations to elucidate the pathophysiological basis for this severe phenotype are warranted.

## Introduction

Neurodevelopmental disorders (NDDs) caused by genetic, epigenetic and environmental factors are clinically heterogeneous conditions affecting the central nervous system in children and include intellectual disability (ID), epilepsy, autism and cerebral palsy. NDDs affect more than 3% of children worldwide. Genetic causes are attributed to variants at over 1,000 loci and include single nucleotide changes (single nucleotide variants, SNV; coding and non-coding variants) and larger losses, gains or rearrangements (structural variants; Tărlungeanu and Novarino, 2018).

Many molecular causes of NDDs disrupt diverse cellular pathways, particularly affecting neuronal development, proliferation and/or migration of cells to cause learning and behavioral disabilities (Srivastava and Schwartz, 2014). One such pathway—that has been the focus of our ongoing investigations—is of the highly conserved TREX (TRanscription-EXport) mRNA export pathway. Multiple TREX complex subunits (e.g., THOC1, THOC2, THOC5, THOC6, and THOC7) have been implicated in NDDs and human disease (Kumar et al., 2015, 2018; Heath et al., 2016; Amos et al., 2017; Mattioli et al., 2019). TREX-mediated mRNA export from the nucleus to the cytoplasm is a complex and highly conserved pathway in all eukaryotes. The TREX complex is composed of a THO sub-complex of a set of six stoichiometric and stable subunits (THOC1-3, 5–7) and accessory proteins (UAP56, UIF, Aly, CIP29, PDIP3, ZC11A, SRRT, Chtop; Heath et al., 2016). THOC subunits are ubiquitously expressed, including in the human brain (Uhlén et al., 2015). The TREX complex has critical roles in developmental processes such as pluripotency maintenance and hematopoiesis as well as in gene regulation, 3′ mRNA processing, stress responses, mitotic progression and genome stability in mammalian cells (Mancini et al., 2010; Yamazaki et al., 2010). We have previously reported genetic and molecular evidence implicating a large number of missense and splicing-defective variants in *THOC2*–which codes for the largest subunit of the TREX mRNA export complex—in NDDs (Mental retardation, X-linked 12/35 MIM 300957; Kumar et al., 2015, 2018). Here we present genetic and molecular evidence on a set of novel *THOC2* variants, and using aggregate data, refine the core clinical phenotype of *THOC2* NDDs.

## Materials and Methods

### Clinical Studies

Through direct contact with clinicians, facilitated by the genotype-phenotype database DECIPHER and the Human Disease Gene Web series, where we moderate a THOC2-related disorder site^1^, 10 individuals from 9 families were identified with rare (absent from gnomAD 2.1) missense variants or an intragenic microdeletion. Eight of these variants were novel and one recurrent (p.Arg77Cys) that was a maternally inherited in individual 8 as previously reported by us (detailed in Supplementary Data; Kumar et al., 2018). In our previous report, we designated p.Arg77Cys as a variant of uncertain clinical significance in the absence of functional studies at the time (Kumar et al., 2018). All families consented to publication of de-identified clinical information, neuroimaging and, for seven families, clinical photographs, in line with local ethics board regulations. The variants have been submitted to ClinVar^2^; accession numbers SCV001132790-SCV001132797.

### Molecular Studies

RNA extraction and RT-qPCR (primers listed in Supplementary Tables S1, S2) were performed as reported previously (Kumar et al., 2015). We used THOC2 Del-Ex37-38 (lymphoblastoid cell lines, LCLs and skin fibroblasts) and p.Asn666Asp (skin fibroblasts) cells from the affected individuals and their carrier heterozygous mothers. However, we used THOC2 p.Arg77Cys and p.Tyr881Cys variant LCLs of only probands. THOC2 Del-Ex37-38 (LCLs and skin fibroblasts) cDNAs (generated by reverse transcribing the total RNAs with Superscript IV reverse transcriptase; Life Technologies, VIC, Australia) were amplified using KAPA HiFi PCR Kit with GC buffer (Kapa Biosystems, IN, USA) and hTHOC2-4326F/P276 and hTHOC3-3′UTR-R2/P392 primers (Supplementary Table S1) at 95°C for 3 min, 35 cycles of 98°C-10 s, 59°C-10 s, 72°C-80 s, incubation at 72°C for 10 min, gel purified (Qiagen MinElute Gel Extraction kit; Qiagen, Victoria, Australia) and Sanger sequenced using the same primers. Genomic deleted region in THOC2 Del-Ex-37-38 carrier mother and affected son was identified by PCR amplification of the target regions from their blood gDNAs using LongAmp Hot Start Taq 2× Master Mix (Promega, Alexandria, NSW, Australia) and hTHOC2-4460-F/P390 and hTHOC2-gDNA-R1/P415 primers (Supplementary Table S1) at 94°C for 30 s, 35 cycles of 94°C-15 s, 64°C-15 s, 65°C-8 min 30 s, incubation at 65°C for 10 min. Appropriate PCR products were gel-purified (Qiagen MinElute Gel Extraction Kit) and Sanger sequenced using hTHOC2-gDNA-F7/P421 and hTHOC2-gDNA-R7/P422 primers (Supplementary Table S1).

### Cellular Studies

We performed THOC2 immunofluorescence staining in Del-Ex37-38 and p.Asn666Asp skin fibroblasts using two anti-THOC2 antibodies; anti-THOC2-I to region between amino acids 1,400–1,450 (Bethyl Laboratories A303-629A, Montgomery, TX, USA) and anti-THOC2-II to a region between amino acids 1543–1593 (Bethyl Laboratories A303-630A) of the THOC2 protein. Both the antibodies were used for detecting the THOC2 protein in Del-Ex37-38 affected individuals and his carrier heterozygous mother fibroblasts but only anti-THOC2-I for detecting the THOC2 p.Asn666Asp in the affected individuals and his carrier heterozygous mother fibroblasts.

### Western Blotting

The Epstein–Barr virus (EBV)-immortalized B-cell lines (LCLs) established from peripheral blood lymphocytes of affected individuals and controls were maintained in RPMI1640 supplemented with 10% fetal bovine serum, 2 mM L-glutamine and 1% Penicillin-Streptomycin at 37°C with 5% CO_2_. Affected individual-derived fibroblasts were cultured in Dulbecco’s modified Eagle’s medium (Sigma) containing 10% fetal bovine serum (Life Technologies), 2 mM L-glutamine, and 1% Penicillin-Streptomycin at 37°C with 5% CO_2_. Total fibroblast proteins were extracted in 50 mM Tris-HCl pH 7.5, 150 mM NaCl, 0.1% Triton-X-100, 1 mM EDTA, 50 mM NaF, 1× Protease inhibitor/no EDTA cocktail and 0.1 mM Na_3_VO_4_ and LCLs in 50 mM Tris-HCl pH 7.5, 5 mM EDTA, 50 mM KCl, 0.1% NP-40 and 1× Protease inhibitor/no EDTA cocktail (Roche protease inhibitor cocktail; Sigma–Aldrich, Castle Hill, NSW, Australia) by low frequency sonication for 12 s and centrifugation at 13,000× *g*. Proteins were assayed using Pierce BCA Protein Assay Kit (Thermo Scientific) according to manufacturer’s protocol. Eight to Ten microgram of protein was resolved on NuPAGE 3–8% Tris-Acetate gel (THOC2) or 8% SDA-PAGE (Laemmli, 1970), transferred to nitrocellulose membranes and western blotted with the following antibodies: anti-THOC1 (Bethyl Laboratories A302-839A), anti-THOC2 (Bethyl Laboratories A303-630A and A303-629A), anti-THOC3 (HPA044009; Sigma–Aldrich, Castle Hill, NSW, Australia), anti-THOC5 (Bethyl Laboratories A302-120A), anti-β-Tubulin (Ab6046; Abcam, Melbourne, VIC, Australia) and polyclonal goat anti-rabbit IgG/HRP (Dako; P0448) as secondary antibody. Signal was detected by Clarity Western ECL Substrate (BIO-RAD 170-5061) and captured using Gel documentation System (BIO-RAD, Gladesville, NSW, Australia).

### *In silico* Pathogenicity Prediction

We used CADD v1.3 (Kircher et al., 2014), SIFT (Sim et al., 2012), Provean (Choi and Chan, 2015), GERP++ (Davydov et al., 2010), PhyloP (Pollard et al., 2010), gnomAD frequency (v2.1.1; Karczewski et al., 2019), Polyphen2 (Adzhubei et al., 2010), MutPred2 (Pejaver et al., 2017), VEST3 (Carter et al., 2013), and Mutation Assessor Score and Pred (Reva et al., 2011) tools for *in silico* prediction of the pathogenicity of different variants (Table 1).

**Table 1:** Pathogenicity predictions and descriptions for the *THOC2* variants.

Variant (NM_001081550.2)	Individual 1 c.229C>T:p. Arg77Cys	Individual 2 c.1996A>G: Asn666Asp	Individual 3 c.2170A>G: p.Lys724Glu	Individual 4 c.2642A>G: p.Tyr881Cys	Individual 5a c.2942G>A: p.Cys981Tyr	Individual 5b c.2942G>A: p.Cys981Tyr	Individual 6 c.3223C>T: p.Arg1075Trp	Individual 7 c.3300G>T: p.Trp1100Cys	Individual 8 c.4646A>G: p.Lys1549Arg	Individual 9 Nullizygosity for the last two coding exons (Ex37-38)
Gender	Male	Male	Male	Male	Male	Male	Male	Male	Male	Male
Mode of inheritance	*De novo*	Mat	*De novo*	*De novo*	Mat	Mat	*De novo*	Mat	*De novo*	Mat
CADD	25.8	26	26.6	26.6	28.3	28.3	27.2	31	22.1	NA
SIFT	Tolerated	Deleterious	Deleterious	Deleterious	Deleterious	Deleterious	Deleterious	Deleterious	Tolerated	NA
Provean score	−3.1	−4.56	−3.6	−5.7	−10.4	−10.4	−7.2	−12.27	−0.52	NA
Provean prediction	Deleterious	Deleterious	Deleterious	Deleterious	Deleterious	Deleterious	Deleterious	Deleterious	Neutral	NA
GERP++	5.09	5.11	5.73	5.73	5.83	5.83	3.02	5.87	4.64	NA
Phylop	2.219	1.69	1.927	2.034	2.458	2.458	0.2	2.479	1.956	NA
gnomAD frequency (v2.1.1)	Absent	Absent	Absent	Absent	Absent	Absent	Absent	Absent	Absent	NA
Polyphen2	P	D	P	D	D	D	D	D	B	NA
MutPred2	0.65	0.843	0.668	0.871	0.95	0.95	0.714	0.955	0.086	NA
VEST3	0.869	0.357	0.864	0.964	0.979	0.979	0.91	0.976	0.168	NA
Mutation Assessor Score	2.39	2.325	2.63	2.855	3.17	3.17	2.67	2.875	0.55	NA
Mutation Assessor Pred	M	M	M	M	M	M	M	M	N	NA
Cell Line available	LCL	Fibroblast	N/A	LCL	N/A	N/A	N/A	N/A	N/A	LCLs and Fibroblasts
Effect of variant on protein	Reduced	Moderately reduced	N/A	Reduced	N/A	N/A	N/A	N/A	N/A	Truncated and increased

*Mat, maternally inherited: D, probably damaging; P, possibly damaging; B, benign; M, Medium; N, Neutral; N/A, not applicable*.

## Results

### Identification of *THOC2* Variants

**Figure 1:**
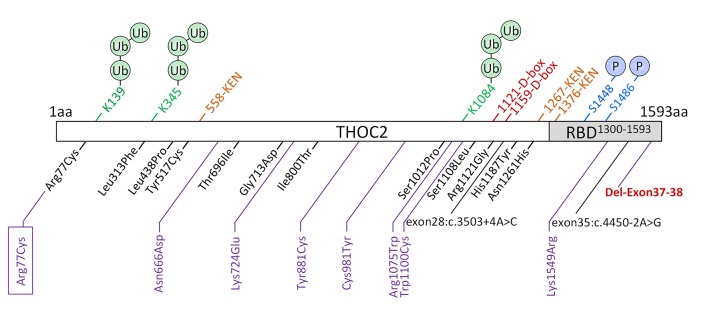
New *THOC2* variants (purple). These include the first Arg77Cys recurrent (boxed) and Del-Exon-37-38 variants (red bold). Previously published *THOC2* variants (black) and structural features are also shown (Kumar et al., 2015, 2018).

**Figure 2:**
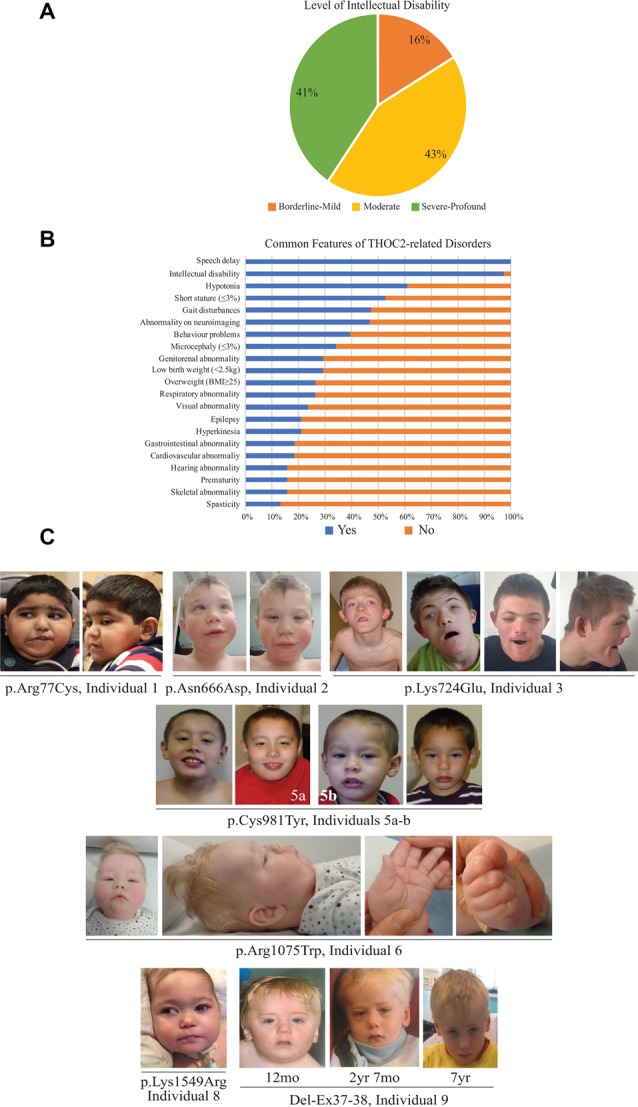
Clinical presentations of the affected individuals identified so far. **(A)** Percentage of individuals with different levels of ID. **(B)** Percentage of individuals with common features. **(C)** Photographs of the affected individuals in the current cohort.

We previously implicated missense and splicing-defective *THOC2* variants in NDDs with a broad range of clinical features (Kumar et al., 2015, 2018). Here we report 10 previously unreported affected individuals with *THOC2* variants, including the first case of a recurrent *THOC2* variant (c.229C>T; p.Arg77Cys). We also identify a novel intragenic *THOC2* microdeletion (Del-Ex37-Ex38) [NM_001081550.2 c.4678–572_4782 + 1215; p.(1559fs16)] (Tables s 1, 2, Figures 1, 2). These variants were identified from either whole exome (WES) or whole-genome sequencing (WGS) of the affected individuals and confirmed by Sanger sequencing of the PCR amplified variant-carrying region from gDNAs of the parents and the affected individuals (detailed in Supplementary Data). The missense *THOC2* variants affect amino acids that are highly conserved (Supplementary Figure S1), are absent in the gnomAD database and are predicted to be pathogenic based on a number of *in silico* analyses tools (Table 1). We have also performed cellular and molecular investigations on variants p.Arg77Cys, p.Tyr881Cys, p.Asn666Asp and Del-Ex37-38, for which we had access to affected individual-derived cells (Table 1; also see below).

**Table 2:** Summary of clinical data of *THOC2* variants.

This Cohort												
Individual Variant details	Individual 1 c.229C>T:p. Arg77Cys	Individual 2 c.1996A>G: Asn666Asp	Individual 3 c.2170A>G: p.Lys724Glu	Individual 4 c.2642A>G: p.Tyr881Cys	Individual 5a c.2942G>A: p.Cys981Tyr	Individual 5b c.2942G>A: p.Cys981Tyr	Individual 6 c.3223C>T: p.Arg1075Trp	Individual 7 c.3300G>T: p.Trp1100Cys	Individual 8 c.4646A>G: p.Lys1549Arg	Individual 9 Nullizygosity for the last two coding exons	Percentage this cohort (*n* = 10)	Percentage total cohort (*n* = 38)
Gender	Male	Male	Male	Male	Male	Male	Male	Male	Male	Male	100% (10/10)	97% (37/38)
Age (years)	6	5	20	5	15	10	2.5	4.5	2	11	2–20 years	2–61 years
Perinatal features												
Prematurity	No	No	Yes	No	No	No	No	No	No	No	10% (1/10)	18% (6/34)
Low birth weight (<2.5kg)	Yes	No	Yes	No	No	No	No	No	Yes	No	30% (3/10)	29% (10/34)
Neurologic features												
Intellectual disability	Yes— Profound	Yes— Profound	Yes— Severe	No	Yes— Severe	Yes— Severe	Yes—Mod+	Yes— Severe	Yes— Severe	Yes—Mod+	9/10 (90%) [22% mod; 78% severe to profound]	97% (37/38)[16% borderline-mild; 27% moderate; 39% severe-profound]
Speech delay	Yes	Yes	Yes	Yes	Yes	Yes	Yes	Yes	Yes	Yes	100% (10/10)	82% (31/38)
Hypotonia	Yes	No	Yes	No	Yes	Yes	Yes	Yes	Yes	Yes	80% (8/10)	61% (22/36)
Spasticity	No	No	Yes	No	Yes	No	No	No	No	Yes	30% (3/10)	13% (5/38)
Hyperkinesia	Yes	No	No	No	No	No	No	No	No	No	10% (1/10)	21% (8/38)
Tremor	No	No	No	No	No	No	No	No	No	No	0% (0/10)	26% (10/38)
Epilepsy	Yes—IS and tonic/myoclonic	No	No	No	No	No	Yes—IS	No	Suspected	Suspected	20% (2/10) confirmed, 20% (2/10) suspected	21% (8/38) confirmed, 8% (3/38) suspected
Gait disturbances	Non ambulatory, hyperkinetic	No	Yes—dystonia, spasticity	No	Yes—poor balance and coordination, weakness, spasticity	Yes—poor balance and coordination, weakness, spasticity	Non ambulatory	Non ambulatory	Non ambulatory	Appendicular spasticity	80% (8/10)	48% (18/38)
Behavior problems	No	No	No	No	No	Yes	No	No	No	No	10% (1/10)	39% (15/38)
Anxiety	No	No	No	No	No	No	No	No	No	No	0% (0/10)	5% (2/38)
Depression	No	No	No	No	No	No	No	No	No	No	0% (0/10)	5% (2/38)
Brain MRI/CT	Delayed myelination, ventriculomegaly, small midbrain	Abnormal CNS myelination	Ventriculomegaly	Normal	ND	Cerebellar atrophy & periventricular changes	Gray matter heterotopia	Delayed myelination, right perisylvian polymicrogyria, short corpus callosum	Ventriculo­ megaly, small midbrain, abnormal cerebellar vermis	Abnormal myelination	89% (8/9)	47% (15/32): 43% ventriculomegaly or cerebral atrophy; 48% white matter abnormality; 19% cerebellar abnormality; 19% abnormality of neuronal migration
Growth parameters												
Microcephaly (≤3%)	Yes	Yes	Yes	No	No	No	Yes	No	No	Yes	50% (5/10)	34% 13/38
Short stature (≤3%)	Yes	No	Yes	No	Yes	Yes	No	No	Yes	No	50% (5/10)	52% 20/38
Overweight (BMI≥25)	No	No	No	No	No	No	No	No	No	No	0% (0/10)	26% (10/38)
Other features												
Visual abnormality	Yes—high myopia	No	Yes—visual impairment	No	No	No	Yes—visual impairment, unilateral coloboma	No	Yes— bilateral exotropia	Yes— blepharophimosis, exotropia	50% (5/10)	9/38 (24%) [ 11% strabismus; 5% nystagmus]
Hearing abnormality	Yes— bilateral OME	Yes—SNHL	No	No	No	No	Yes—mixed	No	No	Yes—	40% (4/10)	6/38 (16%)[8% OME; 8% SNHL]
Genitorenal abnormality	Yes— recurrent UTI		Yes— cryptorchidism	No	No	No	No	No	No	Yes— cryptorchidism	30% (3/10)	10/38 (26%) [19% cryptorchidism]
Gastrointestinal abnormality	Yes—severe feeding difficulties, drooling, GORD : g-tube fed	No	Yes—severe feeding difficulties, GORD	No	Yes— drooling	No	Yes—severe feeding difficulties, GORD	Yes—severe feeding difficulties, chronic constipation	Yes—severe feeding difficulties, oropharangeal dysplasia, drooling, GORD: g-tube fed	Yes—feeding difficulties	70% (7/10)	7/38 (18%) [18% feeding difficulties; 5% dysphagia, 11% GORD, 11% drooling]
Respiratory abnormality	Yes— CLD + oxygen dependency	No	Yes—CLD	No	No	No	Yes— tracheomalacia	Yes— recurrent LRTI	Yes— recurrent apnea and bradycardia,	Yes—recurrent LRTI	60% (6/10)	10/38 (26%) [11% laryngo or tracheomalacia; 5% apnoea; 11% abnormal lung morphology]
Skeletal abnormality	Yes—aquired coxa valga	No	Yes— kyphosis	No	No	No	No	Yes— scoliosis; DDH	No	Yes—scoliosis	40% (4/10)	6/38 (16%)[5% subluxation of the hips and joint laxity]
Cardiovascular abnormaliy	No	No	Yes— abnormal pulmonary artery morphology	No	No	No	Yes— ASD; VSD	Yes— VSD	No	No	30% (3/30)	7/38 (18%)
Other features				Severe speech apraxia with normal IQ.	Dry skin		Cleft palate,		Severe eczema			

*Abbreviations: %, centile; ASD, atral septal defect; VSD, ventricular septal defect; g-tube, gastrostomy tube; mod+, at least moderate severity; GORD, gastroesophageal reflux; DDH, developmental dysplasia of the hip; CLD, chronic lung disease due to recurrent infections; SNHL, sensorineural hearing loss; OME, otitis media with effusionsl; UTI, urinary tract infection; NR, not reported; ND, not done; MRI, magnetic resonance imaging; IS, infantile spasms*.

### Clinical Presentations

The clinical phenotype of affected individuals with *THOC2* variants in this cohort is presented in Table 2 and summarized in Figures 2A,B. The available photographs are shown in Figure 2C. Additional information relating the clinical features is included in case reports in the Supplementary Data and Supplementary Table S2, using an HPO (Human Phenotype Ontology) term framework (Köhler et al., 2019). The main phenotypic features were compared to the individuals reported previously by our group (Table 2, Kumar et al., 2015, 2018). This allowed us to make some general observations about the clinical phenotype of the *n* = 38 individuals with *THOC2* variants (this cohort; Kumar et al., 2015, 2018). First, there is no clear genotypic-phenotypic correlation between the severity or spectrum of phenotypic differences and either the type of genetic variant (missense, splicing-defective or microdeletion) or the part of the protein affected by the variant (Table 2). Second, compared to the first published cohort, which consisted of hemizygous affected males in multi-generational families, the second and this cohort also include many males with *de novo THOC2* variants or *THOC2* variants in siblings which, on further segregation, were shown to be *de novo* in the heterozygous mother. The initial cohort described males with typically mild to moderate ID. Of 20 individuals, 30% (6/20) had borderline-mild ID, 55% (11/20) moderate ID and only 15% (3/20) severe ID (Kumar et al., 2015). In contrast, our last (Kumar et al., 2018) and current cohort includes a higher proportion [75% this cohort; 62.5% (Kumar et al., 2018)] of males with severe to profound ID. In striking contrast to our overall findings is individual 4 with the p.Tyr881Cys variant. This male, in whom pathogenicity of p.Tyr881Cys variant is supported by evidence from functional studies (Figures 5B,C: detailed results below), is unique in our cohort as having no evidence of general cognitive dysfunction. On psychometric testing, he had a performance IQ of 104, within the normal range. He did have significant language disorder, with a formal diagnosis of speech apraxia. At the age of 5, he could effectively communicate by sign language. He has a history of mild gross motor delay. Therefore, this publication clarifies that the range of cognitive disability in THOC2-related disorder is very broad—ranging from a normal IQ to a profound ID (Figure 2A).

**Figure 3:**
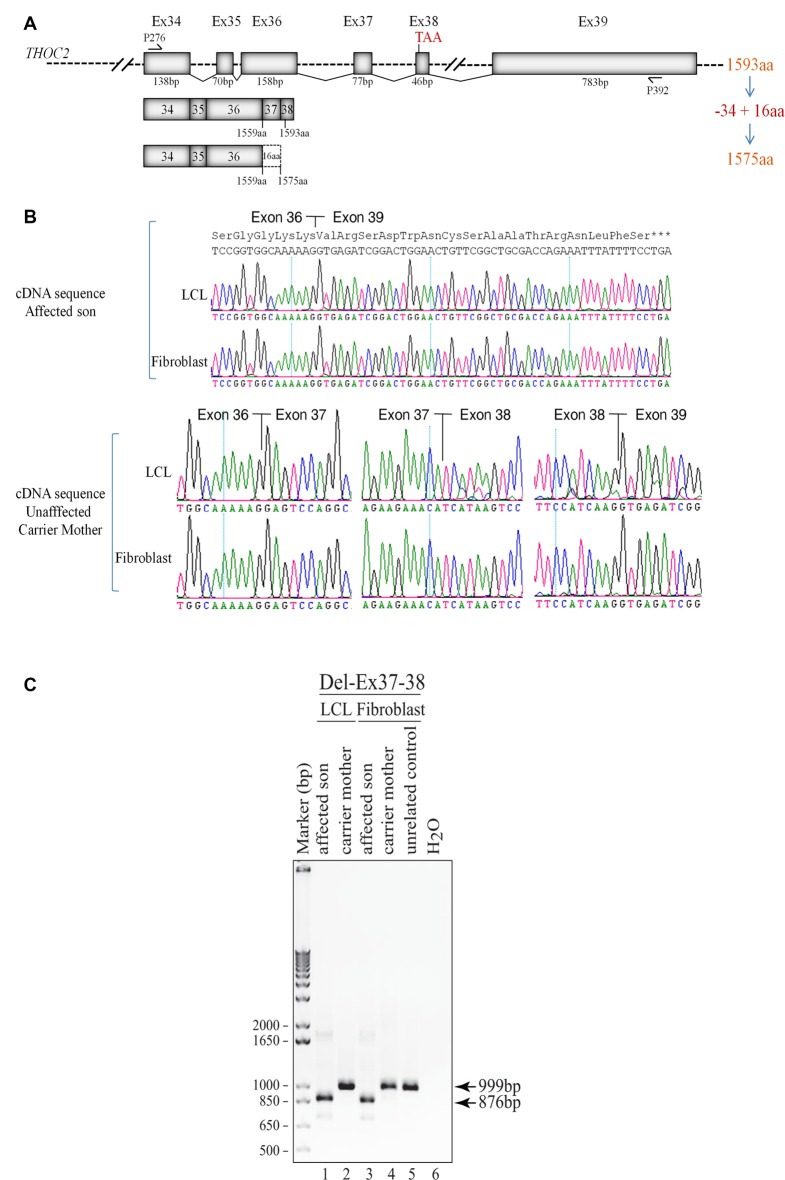
Del-Ex37-38 results in the loss of 34 C-terminal amino acids in the affected individual. **(A)** Part of the *THOC2* gene showing Ex34 to Ex39 and positions of primers. Ex37-38 deleted mRNA translates a 1575 amino acid protein, lacking 34 C-terminal amino acids but adding 16 amino acids encoded from the 3′ UTR region of the deleted mRNAs. **(B)** Sanger sequencing chromatograms of DNA amplified from LCL and fibroblast cDNAs of the affected individual and his heterozygous unaffected carrier mother using primers P276/P392 located within Ex34 and Ex39. Wild type (1593 amino acid) and C-terminal deleted THOC2 protein (1575 amino acids) translated from Del-Ex37-38 mRNA is also shown. **(C)** Ex37-38 coding sequence is deleted in both fibroblast and LCL THOC2 mRNAs of the affected son but not in the unaffected carrier mother. Total fibroblast and LCL RNAs were reverse transcribed and PCR amplified using primers P276/P392. PCR products (999 bp from the carrier mother and 876 bp from the affected son) were gel purified and Sanger sequenced using P276 and P392 primers.

**Figure 4:**
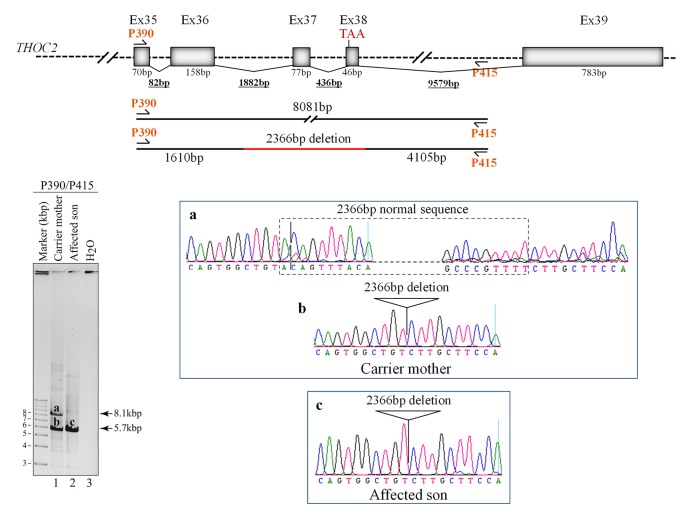
Del-Ex37-38 results from the deletion of approximately 2.4 kb genomic DNA (chrX:122743574-122745939) flanking the Ex37-38. Sequences around the deleted target region were amplified from the affected son and carrier mother’s blood gDNA using P390/P415 primers using LongAmp Hot Start Taq 2× Master Mix, resolved on agarose gel and bands a-c were eluted and Sanger sequenced. Positions of the P390/P415 primers and sequencing chromatograms (a–c) around the deleted gDNA regions are shown.

**Figure 5:**
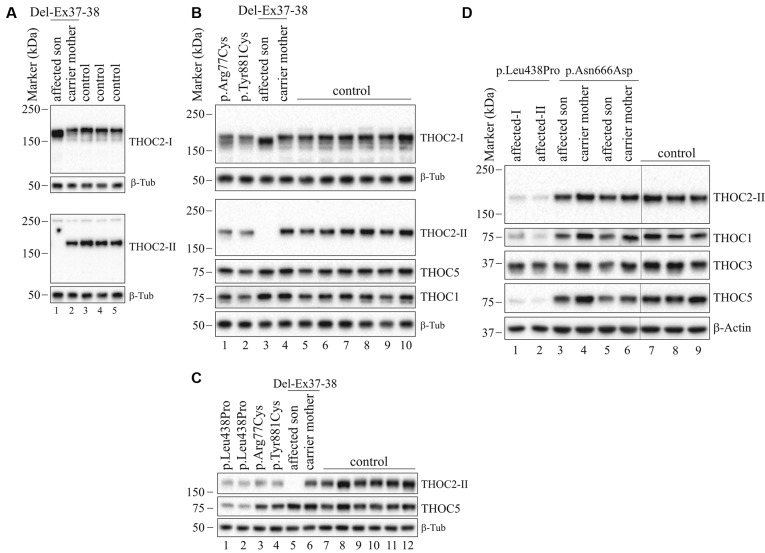
THOC2 variant protein analysis in patient-derived cell lines. **(A)** THOC2 protein in affected son with Del-Ex37-38, unaffected carrier mother and control fibroblasts. Total protein lysates were western blotted with an anti-THOC2-I antibody that binds to a region between amino acids 1,400–1,450 coded by Ex32-34 mRNA (upper panel) and anti-THOC2-II antibody that binds to a region between amino acids 1543–1593 coded by Ex37-38 mRNA (lower panel). Samples were probed for β-Tubulin as a loading control. **(B)** THOC2 protein in p.Arg77Cys, p.Tyr881Cys, affected son with Del-Ex37-38, unaffected carrier mother and control LCLs. Total protein lysates were western blotted with anti-THOC2-I, anti-THOC2-II, anti-THOC1, anti-THOC5 and anti-β-Tubulin (loading control) antibodies. Del-Ex37-38 affected fibroblasts (Lane 1 in panel **A**) and LCLs (Lane 3 in panel **B**) showing presence of a C-terminally deleted smaller but higher levels of the THOC2 protein when western blotted with anti-THOC2-I antibody (upper panels) that is absent in the affected fibroblast lysates probed with anti-THOC2-II antibody (lower panels). However, normal full-length THOC2 protein is present in the carrier mother and controls western blotted with both the anti-THOC2-I and anti-THOC2-II antibodies (Lane 2 in upper and lower panels in **A** and Lane 4 in upper and lower panels in **B**). The p.Arg77Cys and p.Tyr881Cys THOC2 levels are reduced. **(C)** THOC2 protein levels in p.Leu438Pro (reported to have reduced protein stability; Kumar et al., 2015), p.Arg77Cys, p.Tyr881Cys, Del-Ex37-38 affected son, his unaffected carrier mother and control LCLs. Total protein lysates were western blotted with anti-THOC2-II, anti-THOC5 and anti-β-Tubulin (loading control) antibodies.** (D)** The p.Asn666Asp levels are moderately reduced in the affected fibroblasts. p.Asn666Asp affected son, his unaffected carrier mother and control fibroblast total lysates were western blotted with the antibodies as shown. p.Leu438Pro fibroblasts previously reported having reduced THOC2 protein stability were included as controls (Kumar et al., 2015). Vertical lines on western blot images indicate the site of deleted sample lanes.

Third, data in Figure 2B and Table 2 suggest that THOC2-related disorder should be considered a multi-systemic disorder in a significant proportion of individuals. The most common extra-neurological feature is disorders of growth. Growth disorders are common across the whole expanded cohort (Figure 2B); a half (52%) have persistent short stature, a third (34%) are microcephalic and 29% have intrauterine growth restriction (IUGR). There was no relationship between IUGR in the offspring and carrier status in the mother. 26% of the expanded cohort have increased body weight, with obesity in older males particularly noted. Congenital anomalies of the cardiorespiratory, genitorenal and skeletal systems, severe feeding difficulties and gastroesophageal reflux, and visual and hearing impairments are present in a significant proportion (10–30%). Therefore, comprehensive evaluation including detailed systems review by a pediatrician, cardiac echocardiogram, hearing screening and ophthalmological review in all newly diagnosed patients is highly recommended. Photographs where available are shown in Figure 2C. As was the case in our previous publications, an easily recognizable facial gestalt is not apparent, although children with a more severe phenotype (Individuals 1, 6, 8 and 9) have downturned corners of their mouth and deep-set almond-shaped eyes: a facial appearance, which may reflect reduced tone.

Fourth, a more complex neuromuscular phenotype is emerging in a proportion of the cohort: that of severe to profound ID, persistent muscular hypotonia, recurrent aspiration and respiratory tract infections, excessive salivation and an increased risk of congenital anomalies, including laryngo/tracheomalacia, cardiac and palatal anomalies. This phenotype was present in the individual with p.Arg77Cys variant for which initial functional studies using exogenous THOC2 variant expression in HEK293T cells were not conclusive (Kumar et al., 2018). We classified this as a variant of uncertain significance, also in view of this patient’s phenotype, which was quite different from the majority of the cohort. We are now more confident that this variant is pathogenic. The variant is recurrent and *de novo* in individual 1 in this cohort and molecular studies from patient-derived cell lines available from this second family are consistent with reduced protein levels. Both individuals with the p.Arg77Cys variant has strikingly overlapping phenotypes (see Supplemental Data for detailed case reports). Individuals 6, 8 and 9 from the current cohort also have similarities to this phenotype. This more severe phenotype is also frequently associated with neuroradiological abnormalities and an increased chance of a seizure disorder. Seizure disorder, although not very common over the whole cohort (Figure 2B: 21%), can be problematic – with a severe developmental and epileptic encephalopathy picture, commonly including infantile spasms. Therefore, the clinicians should have a low threshold for investigating neurological symptoms or signs with an EEG and MRI brain.

Fifth, although for many individuals the phenotype is static, a more complex neurological phenotype also emerges with age in several males; in the expanded cohort 13% develop hypertonia and spasticity or contractures, and 53% have some abnormality of motor coordination, stereotypic or involuntary movements. In the current cohort, three individuals had received an additional diagnosis of cerebral palsy. Of the nine affected individuals included in this cohort, cases 1, 4, 5, 6, 7 have had multi-timepoint MRI neuro-imaging reviewed by a single Neuroradiology Attending Consultant with dedicated pediatric expertise (*data not shown*). Although no unifying neuroradiological abnormalities were delineated from this small cohort, one affected individual demonstrated interval cerebellar hemispheric volume loss after a 6-year period, and another demonstrated volume loss of the corpus callosum and mild reduction in myelination at an early post-natal 4-month scan. A further patient had reported features of nodular heterotopia although this imaging was not available for review. No other definitive migrational anomaly, midline defect or development abnormality was identified. The potential implications on white matter development and degeneration in this cohort require long term follow-up imaging.

Lastly, all heterozygous female carriers in this cohort had normal intelligence, as was the case for the entire cohort, with the exception of the one female reported to date with a *de novo* *THOC2* variant with a developmental and epileptic encephalopathy (Kumar et al., 2018). When tested, asymptomatic female carriers are highly skewed for X inactivation (Kumar et al., 2015, 2018).

In summary, this expanded cohort has clarified that the core phenotype of THOC2 related disorder in hemizygous males is that of language disorder and ID, with a wide variability in severity, and that disorders of growth are common, and multi-systemic involvement is present in a significant proportion. A subpopulation of patients has a very severe phenotype with severe-profound ID, persistent hypotonia, respiratory abnormalities and a higher chance of other congenital anomalies. Some affected males show the progression of neurological symptoms and progressive neuroradiological features. Further research to explore the underlying pathophysiological basis for these more severe phenotypes is warranted, but likely reflect the involvement of THOC2 in multi-organ development and function.

### Molecular Studies

We used primary skin fibroblasts and/or immortalized B-lymphocytes (LCLs) derived from affected individuals and where available, also their heterozygous carrier mothers.

### Del-Ex37-38 THOC2 Variant

**Figure 6:**
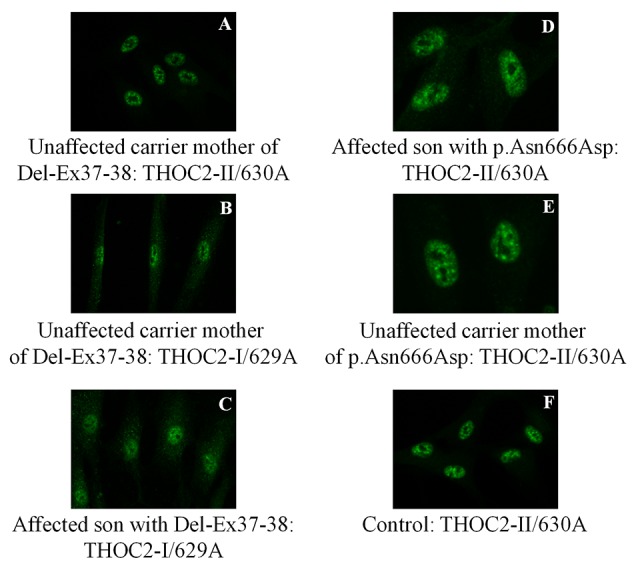
THOC2 localization is unaltered in variant fibroblasts. THOC2-II antibody detects THOC2 protein in fibroblasts of the Del-Ex37-38 unaffected carrier mother **(A)** but not affected son (not shown, as we detected no fluorescence signal). However, the THOC2-I antibody detects THOC2 protein in fibroblasts of unaffected carrier mother **(B)** and the affected son with Del-Ex37-38 **(C)**. Localization p.Asn666Asp THOC2 protein in fibroblasts of an affected son **(D)**, his unaffected carrier mother **(E)** and control **(F)**.

**Figure 7:**
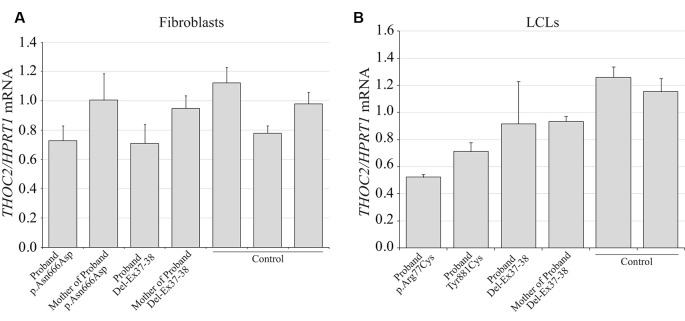
THOC2 mRNA expression in the variant cell lines. cDNA was generated by reverse transcribing the total RNA extracted from variant fibroblasts **(A)**, LCLs **(B)** and appropriate controls using SuperScript IV reverse transcriptase and assayed for *THOC2* expression (relative to *HPRT1* housekeeping gene) with SYBR green master mix and primer pairs listed in Supplementary Table S1. Assays were performed two times independently and error bars show SDs.

We found an affected male with deletion of *THOC2* Ex37-38 (NM_001081550.2) as identified by massively parallel sequencing (see Figure 3). We have subsequently validated this as a 2.4 kb X-chromosome gDNA microdeletion (encompassing Ex37-38 sequence) which was also present in his carrier mother (Figure 4). In addition, we PCR amplified the Ex35-Ex39 coding region from cDNAs prepared by reverse transcribing the LCL and fibroblast mRNAs from the affected individual and his normal carrier mother (Figure 3C). Sanger sequencing of the amplified products showed deletion of Ex37-38, but not Ex39 coding sequences, in the affected individual (Figures 3B,C). The carrier mother’s cells showed mRNAs with Ex37-38 sequences consistent with her X-inactivation skewing (10:90; Figures 3A–C). We detected a slightly smaller THOC2 protein (due to Ex37-38-encoded C-terminal 34 amino acid deletion) in affected son’s LCLs and fibroblasts but normal full-length THOC2 protein in the unaffected carrier mother on western blots probed with anti-THOC2-I antibody that binds to a region between amino acids 1,400–1,450 coded by Ex32-33 mRNA sequences (compare lanes 1 and 2, upper panel, Figure 5A and lanes 3 and 4, Figure 5B). Notably, C-terminal THOC2 truncated protein levels were higher in both the affected LCLs and fibroblasts (Figures 5A,B). As expected, the anti-THOC2-II antibody that binds to a region between amino acids 1543–1593 coded by Ex37-38 mRNA sequences (lower panel) did not detect the THOC2 protein in affected son’s LCLs and fibroblasts that express a C-terminally-deleted THOC2 protein. This antibody however detected THOC2 protein in the carrier mother-derived cells as she expressed normal full-length THOC2 protein (compare lanes 1 and 2, lower panel, Figure 5A and lanes 3 and 4, Figure 5B). THOC1 and THOC5 levels were similar in fibroblasts of the affected son and carrier mother (Figure 5A). Likewise, anti-THOC2-I antibody detected nuclear THOC2 protein in both the affected son and carrier mother’s fibroblasts by immunofluorescence but anti-THOC2-II antibody, as expected, detected the THOC2 protein in the mother’s but not son’s fibroblasts (Figures 6A–C). Normal nuclear THOC2 localization was detected in fibroblasts of the affected son and carrier mother (Figure 6F). We observed no significant difference in *THOC2* mRNA level in LCLs and fibroblasts of the affected son and carrier mother (Figure 7A).

### THOC2 Missense Variants

LCLs were available from affected individuals’ hemizygous for the p.Arg77Cys and p.Tyr881Cys variants and fibroblasts from the affected individual and his carrier mother with the Trp.Asn666Asp variant (Figure 5). p.Arg77Cys and p.Tyr881Cys THOC2 variants resulted in reduced protein levels compared to control LCLs (Figures 5B,C). THOC1 and THOC5 levels were also moderately reduced in these LCLs (Figures 5B,C). Interestingly, a reduction in p.Arg77Cys and p.Tyr881Cys THOC2 variant protein levels were similar to unstable p.Leu438Pro variant THOC2 protein reported previously (Figure 5C; Kumar et al., 2015). Note that RT-qPCR assays in p.Arg77Cys and p.Tyr881Cys THOC2 variant LCLs also showed reduced mRNA expression compared to unrelated normal controls (Figure 7B). Western blotting on p.Asn666Asp fibroblasts also showed a small but reproducible reduction in THOC2 protein and related THO complex subunits THOC1, THOC3 and THOC5 compared to his unaffected carrier mother and unrelated normal controls (Figure 5D). p.Asn666Asp THOC2 reduction was much less pronounced than that of highly unstable p.Leu438Pro THOC2 as well as THOC1 and THOC5 proteins in affected fibroblasts (Figure 5D). p.Asn666Asp THOC2 localization was not affected (Figures 6D,E). Levels of mRNA expression in p.Asn666Asp affected LCLs were similar to that of the patient’s carrier mother and unrelated controls (Figure 7A).

## Discussion

TREX-mediated mRNA export is a fundamental process that is essential for the export of mRNA from the cytoplasm to the nucleus to allow translation of the normal level of protein required for efficient growth and development. We and others have presented evidence showing that perturbed mRNA export leads to altered cellular function, thus causing a range of diseases (Di Gregorio et al., 2013; Kumar et al., 2015, 2018; Heath et al., 2016; Mattioli et al., 2019). During the last few years, we have focussed on the association of variation in the *THOC2* gene and NDDs. *THOC2* is intolerant to complete loss of function [probability of loss of function or pLI = 1; o/e = 0.02 (0.01–0.08): gnomAD v.2.1.1]. We first reported four *THOC2* missense inherited variants in the affected individuals with variable degrees of ID and commonly observed features such as speech delay, elevated BMI, short stature, seizure disorders, gait disturbance, and tremors (Kumar et al., 2015). In 2018, we reported an additional six affected individuals from five unrelated families with two *de novo* and three maternally inherited pathogenic or likely pathogenic missense and splicing-defective *THOC2* variants. The study also included a *de novo* variant in a female with epileptic encephalopathy. We observed a core ID phenotype and common behavioral features, infantile hypotonia, gait disturbance and growth impairment (Kumar et al., 2018). In the current cohort, we present molecular and clinical findings on ten additional *THOC2* variant individuals, confirming a more severe THOC2-related phenotype associated with severe hypotonia, respiratory and other congenital anomalies, and summarize the phenotypic impact of all *THOC2* variants to date in 38 individuals. This publication, therefore, may serve as the most up to date reference of this X-linked NDD condition.

Variants in TREX subunit proteins can alter their stability (Kumar et al., 2015, 2018; e.g., THOC2 ID variants), interaction (e.g., THOC6 syndromic ID variants; Mattioli et al., 2019) and/or localization (e.g., THOC6 syndromic ID variants; Mattioli et al., 2019). We observed reduced levels of p.Arg77Cys THOC2 (although the stability of this variant exogenously expressed in HEK293T cells was uninformative, see Figure 3 in Kumar et al., 2018) and p.Tyr881Cys THOC2 in the affected LCLs and p.Asn666Asp THOC2 affected fibroblasts (without THOC2 mislocalization). Variant THOC2 protein reduction also impacted the THOC1, THOC3 and THOC5 stability in the variant cells, which is consistent with a number of reports showing that instability of THO subunits—either by missense changes or siRNA-mediated knockdown—results in reduced levels of other THO subunits (Chi et al., 2013; Kumar et al., 2015, 2018). As the *THOC2* mRNA levels in the affected LCLs or fibroblasts were comparable to the controls, the reduced THOC2 variant protein levels are most likely due to enhanced proteasome-mediated degradation of ubiquitinated THOC2 (Lopitz-Otsoa et al., 2012). Reduced or depleted THO subunit proteins have been shown to cause severe to mild nuclear mRNA retention (Chi et al., 2013) and hence can cause variable molecular and cellular consequences both in mammalian cells and lower organisms. For example, THOC2 depletion impacts chromosome alignment, mitotic progression, and genomic stability in human HeLa cells (Yamazaki et al., 2010). Thoc2 knockdown in *Drosophila* S2 cells inhibits cell proliferation and heat-shock mRNA export (Rehwinkel et al., 2004). Thoc2 depletion results in a significant increase in the length of neurites in cultured rat primary hippocampal neurons and *C. elegans* *thoc2* knockout worms are slow-growing, sterile, have functional defects in specific sensory neurons and die prematurely (Di Gregorio et al., 2013). *Danio rerio Thoc2* is essential for embryonic development (Amsterdam et al., 2004). Interestingly, a *de novo* translocation creating a *PTK2-THOC2* fusion that reduced the expression of the two genes in a female patient was implicated in cognitive impairment and cerebellar hypoplasia (also reported in 4/19 of our cases; Di Gregorio et al., 2013). THOC2 or THOC5 depletion leads to dedifferentiation of vascular smooth muscle cells, characterized by increased migration and proliferation (Yuan et al., 2018). Thoc2 and Thoc5 depletion was shown to cause retention of self-renewal (Oct4) and pluripotent gene transcripts (*Nanog*, *Sox2*, *Esrrb*, and *Klf4*) as well as increased differentiation gene expression (*Cdx2*, *Gata3* and *Gata4*) in mouse embryonic stem cells, resulting in a decrease in cell pluripotency and proliferation (Wang et al., 2013).

We observed accumulation of 34 C-terminal amino acid THOC2 truncated protein in Del-Ex37-38 LCLs and fibroblasts that is similar to the accumulation of 110 C-terminal amino acid deleted THOC2 protein in fibroblasts of the affected individual carrying the exon35:c.4450–2A>G splice-variant and potential loss of the 1,300–1,593 amino acid putative RNA binding domain (RBD) in a proportion of blood cells with the exon28:c.3503 + 4A>C defective splice-variant (Kumar et al., 2018). The truncated proteins in these individuals had normal nuclear localization. Together, the affected individuals (at least in Del-Ex37-38 and exon35:c.4450–2A>G splice-variant) not only have truncated THOC2 protein (where a part of the RBD is lost) but actually more of it, which may indicate presence of a degradation signal within this region and/or stabilization due to structural changes to the THOC2 protein that may or may not be impacting interactions with other TREX subunits. That C-terminally truncated THOC2 protein potentially has perturbed function is consistent with yeast Tho2 studies showing that Tho2Δ1408–1597 (small C-terminal RBD deletion) grows slower than the wild-type strain and ThoΔ1271–1597 (complete RBD deletion) strain barely grows at restrictive temperature (Peña et al., 2012).

THOC2 depletion leads to almost complete retention of mRNAs in the cell nucleus, suggesting that it is an essential mRNA export factor whose complete knockout can be potentially toxic to the cell (Chi et al., 2013). We, therefore, suggest that all the identified *THOC2* variants result in partial loss-of-function that alters normal mRNA export in neuronal and likely other cells, thereby causing a broad range of clinical presentations in the affected individuals carrying the *THOC2* variants. However, although THOC2 depletion causes bulk mRNA nuclear retention in HeLa cells (Chi et al., 2013), whether *THOC2* variants affect—bulk or specific—mRNA export in neuronal cells has not been elucidated. TREX complex was originally thought to be a bulk mRNA export pathway but a number of reports have shown its role in specific mRNA export. For example, the Thoc1/2 and Thoc5 or Thoc6 are responsible for nuclear export of only a subset of mRNAs (e.g., heat shock mRNAs) in Drosophila and mammalian cells, respectively (Rehwinkel et al., 2004; Katahira et al., 2009; Guria et al., 2011). Thoc2 and Thoc5 selectively bind and regulate the export of mRNAs involved in the maintenance of pluripotency (e.g., *Nanog*, *Sox2*, *Esrrb*, and *Klf4* mRNAs (Wang et al., 2013) and Thoc5 in hematopoiesis (Mancini et al., 2010).

The published data in conjunction with our molecular and cellular data, and broader clinical presentations in our cohort indicate that THOC2 reduction (and consequently other THO subunits) or potential alterations in TREX subunit interactions due to intragenic deletion of C-terminal RBD and/or structural interactions as a result of THOC2 missense changes result in perturbed mRNA export, causing cellular dysfunction and NDDs. This view is supported by the published evidence showing that slight perturbations in mRNA export can lead to NDDs (Beaulieu et al., 2013; Kumar et al., 2015, 2018; Mattioli et al., 2019), neurodegenerative disease (Woerner et al., 2016) and cancer (Domínguez-Sánchez et al., 2011). There are also examples showing how variants in the human mRNA export mediator GLE1 result in a severe fetal motor neuron disease (Nousiainen et al., 2008) and amyotrophic lateral sclerosis (ALS; Kaneb et al., 2015), and toxic CUG expansion in the 3′ untranslated region of the DM protein kinase mRNA can cause myotonic dystrophy type I by impairing RNA transport out of the nucleus (Brook et al., 1992).

Based on clinical and, where available, molecular data on 38 individuals, we have come to a realization that the THOC2-linked NDDs can present with highly variable phenotypes. Intriguingly, we also identify a case of severe expressive dysphasia without obvious intellectual impairment. This particular case and observation still awaits replication and as such further validation. In hindsight, it is perhaps not surprising that even subtle perturbation to a crucial cellular mechanism like TREX mRNA export can yield complex and often variable clinical presentations which can not only be driven by the *THOC2* gene variation itself, but can also be modified by genome-wide polygenic risk (Niemi et al., 2018) or other yet to be identified factors.

## Data Availability Statement

The datasets have been uploaded to ClinVar and are available here: https://www.ncbi.nlm.nih.gov/clinvar/ SCV001132790[clv_acc] under accession numbers SCV001132790, SCV001132791, SCV001132792, SCV001132793, SCV001132794, SCV001132795, SCV001132796 and SCV001132797.

## Ethics Statement

The studies involving human participants were reviewed and approved by WCHN Human Research Ethics Committee and University of Adelaide, Adelaide. Written informed consent to participate in this study was provided by the participants’ legal guardian/next of kin. Written informed consent was obtained from the minor(s)’ legal guardian/next of kin for the publication of any potentially identifiable images or data included in this article.

## Author Contributions

RK, EP, MF, and JG designed the experiments and wrote the article. RK performed the molecular studies. AEG helped with pathogenicity predictions. RC with tissue culture. SB, OA, DD, YE, CC, AG, MS, RM, LB, MT, DNA, VA, MB, GB, LD, RH, CM, GC, MW, and JB contributed THOC2 variants, photos, clinical data, and in some cases, the patient-derived cells for molecular investigations. JD reviewed MRI neuro-images. EP and ML collated the clinical data. All authors read and made suggestions on the manuscript.

## Conflict of Interest

The authors declare that the research was conducted in the absence of any commercial or financial relationships that could be construed as a potential conflict of interest.

## Footnotes

^1^https://humandiseasegenes.nl/thoc2/

^2^https://www.ncbi.nlm.nih.gov/clinvar/?term=“GOLD%20service%2C%20Hunter%20New%20England%20Health”[submitter]+AND+“THOC2”[gene]
