# Supplementary material for: Expanding Clinical Presentations Due to Variations in THOC2 mRNA Nuclear Export Factor
Source: Front Mol Neurosci. 2020 Feb 11;13:12. doi: 10.3389/fnmol.2020.00012 (PMC7026477; doi:10.3389/fnmol.2020.00012)
Supplement: Supplementary file 3 [file Data_Sheet_1.docx]

**SUPPLEMENTARY MATERIAL**

**Expanding clinical presentations due to variations in THOC2 mRNA nuclear export factor**

**Kumar et al**

**Detailed Clinical Data**

**Patient 1: *De novo* pArg77Cys**

This 6-year-old male was delivered by caesarean section at 38 weeks for breech presentation. Apgar scores were 9 at 1 and 10 at 5 minutes. He was admitted into the NICU for the first few days for poor feeding. Birth weight was 2.37kg. Intrauterine growth restriction was detected prenatally and there were concerns with decreased fetal movements.

At 5 years 10 months his weight was on the fifth centile, height <1^st^ centile, and head circumference <2^nd^ centile. He has profound intellectual disability (ID). He sat at 24 months and stood without support at 30 months. He has a rake grasp. He cannot walk independently. He had no words at 5 years. He does not smile but recognises her parents. He has persistent poor central muscular tone. Electromyography and nerve conduction studies were within normal limits at 16 months. He has bilateral coxa valga deformity. He has epilepsy: infantile spasms and myoclonic seizures. He has abnormal hyperkinetic movements.

First MRI was at 18 months: he has delayed myelin maturation, small midbrain and non-specific areas of white matter hyperintensity on T2 weighted hyperintensity. Repeat MRI at 4 years showed interval development of a bright signal intensity involving the olives of medulla oblongata, a droopy appearance to the corpus callosum and a small midbrain. On spinal images, he has a low-lying conus and transitional vertebra in the lumbosacral region.

He has persistent feeding dysfunction requiring gastrostomy tube feeding and gastrooesphageal reflux managed with domperidone and omeprazole. He has not been fed orally since 6 months of age. He has multi-pathogen colonisation of his gastrointestinal tract.

He has recurrent respiratory infections due to repeated aspirations with evidence of bronchiectasis. He has laryngomalacia. He has low oxygen. He has excessive salivation. Echocardiogram was normal.

He has recurrent urinary tract infections and urinary retention requiring silodosin and daily intermittent catheritisation.

He has high myopia, visual impairment, bilateral mild elevation of the optic nerve. He required bilateral myringotomy and tube insertion for recurrent otitis media with effusions.

**Individual 2: Maternally inherited p.Asn666Asp**

This is a 5 year old male who was born at 39 weeks gestation with some respiratory distress requiring brief positive pressure ventilation but no invasive ventilation or admission to the neonatal intensive care unit. Apgar score were 7 at 1, 5 at 5 minute and 9 ant 10 minutes. His birth weight was 3.45 kg and length was 49.5 cm. His current head circumference is at the 3^rd^ percentile, weight at the 30-40^th^ percentile.

He has severe to profound ID. He has possible Attention deficit hyperactivity disorder but this has not been formally diagnosed. An MRI of the brain at 5 years of age demonstrated mild delay in myelination (incomplete myelination of sub-cortical U-fibers), and mild cerebellar and anterior corpus callosum atrophy.

He has bilateral sensori-neural hearing loss and has dysmorphic features including; epicanthal folds, flattened nasal bridge, low set ears, mild dolichocephaly and microcephaly. He has mild clinodactyly of great toes and upturned nails.

**Individual 3: *de novo* p.Lys724Glu**

This individual is 20 years old. He was delivered at 32 weeks by emergency caesarean section. The pregnancy was complicated by IUGR, oligohydramnios, maternal HELLP and pre-eclampsia. He required resuscitation and intubation at birth. Apgars were 5 at 1 minute and 9 at 5 minutes. Birth weight was 1.05 kg.

He developed respiratory distress syndrome, which developed into chronic lung disease, and oxygen dependence for the first year of life. He was admitted for 10 weeks in the NICU. He required oxygen for the first year of life. He has had recurrent lower respiratory infections and asthma and has chronic lung disease.

He required orogastric feeding for the first 7 weeks of life and then was transitioned to thickened fluids and solids.

He has severe developmental delay with further regression of skills at 2 years of age. He has poor central muscular tone with peripheral spasticity. He had a marked startle reflex on examination at 9 years. He holds his hands together and puts his hands repetitively in his mouth and wrings them. He rocks and chews his fingers. He started to vocalise at 11 years of age and cries to express his needs. He makes noises and laughs on contact with other people. He has persistent poor central muscle tone.

He has had two MRI brain scans: on the first he was noted to have an abnormal shape to his skull and brain. On the second, it was noted he had mild dilatation of his third and lateral ventricles.

He has stenosis of the pulmonary valve and right main pulmonary artery. He has cryptorchidism. He has delayed visual development and wears glasses. He has skeletal kyphosis.

He has dysmorphic features including delayed eruption of permanent teeth, a low anterior and posterior hairline, mandibular prognathia, flat facial profile with small prominent ears. He has slight hypertelorism, down-slanting palpebral fissures and arched eyebrows. He has a long thin upper lip, downturned corners of the mouth, a smooth philtrum, coarse hair, generalised hirsutism, and had premature adrenarche.

**Individual 4: *De novo* p.Tyr881Cys**

This individual, now 5 years of age, was born at 41 weeks with a birth weight of 4.2kg and normal Apgar scores. He required support for feeding in the first week of life due to poor sucking. Growth measurements are normal, with a head circumference on the 50-75^th^ centile, height on the 25^th^ centile and weight on the 50^th^ centile.

His speech and language development were severely delayed and at the age of 4 years he was diagnosed with speech apraxia. Currently he speaks a few single words and is proficient in sign language. He has mild motor delay with mild abnormality in the regulation of tone. Cognitive development was assessed using the Snijders-Oomen Nonverbal revised intelligence scale for young children (Tellegen P. J., 1998; Thompson et al., 2010) a non-verbal intelligence scale suitable for all children from age 2 years 3 months to 7 years 3 months, which is particularly suited for children with hearing impairments and/or language or verbal communication deficits. Total IQ score was 114, with a Performance Scale IQ score of 104 and a Reasoning Scale IQ of 122. A MRI brain scan showed no abnormalities.

On physical examination, some dysmorphism was observed including mild retrognathia, prominent ears and 2-3 toe syndactyly.

**Individual 5a and 5b: Maternally inherited p. Cys981Tyr**

These are two brothers 10 and 15 years of age.

**Individual 5a** is 15 years of age. He was delivered at term. Birth weight was 3.03kg.

Currently his head circumference is on the 50^th^ centile, height between the 1^st^ and 3^rd^ centile and weight on the 90^th^ centile.

He has a severe ID. He has abnormal tone with low central tone progressing to peripheral spasticity, poor balance and coordination, and an abnormal ‘crouched’ gait. He has excessive salivation.

He has dry skin and acanthosis nigricans.

He has mild dysmorphic features, a right epibulbar dermoid, mild ptosis, persistent epicanthal folds, mild micrognathia, a tented upper lip and widely spaced teeth. He has distally tapered fingers and a hockey stick crease.

**Individual 5b** is 10 years old. He was born at 40 weeks. Birth weight was 3.8kg, head circumference was 36.8c, and length 54cm.

At 10 years of age, his weight is on the 1^st^ centile, height 3^rd^-10^th^ centile and head circumference <2^nd^ centile.

He has a severe ID, infantile hypotonia and weakness. He has progressive spasticity, and, similar to his brother he has an unusual crouched gait. He has aggressive behaviour.

On brain MRI, there is evidence of cerebellar atrophy and periventricular changes.

He has mild dysmorphic features including ptosis, mild micrognathia, periorbital fullness and widely spaced teeth. He has distally tapering fingers and a right transverse palmar crease.

**Individual 6: *De novo* p.Arg1075Tyr**

This male is 2 years 6 months. He was delivered at term after an uncomplicated pregnancy. He was admitted to NICU after birth with respiratory distress and required supplementary oxygen for the first three days of life. Birth weight was 3.4kg.

He required initial support with feeding with nasogastric tube feeding. His head circumference was <2^nd^ centile, length was on the 1^st^ centile and weight between the 3^rd^ and 10^th^ centile. He has persistent feeding difficulties in the first year of life with gastrooesophageal reflux, which improved on ranitidine. He now tolerates soft mashed food.

He has a moderate global developmental delay: at one year of age he was rolling, sitting briefly unsupported, reaching for objects and was making no clear babble. He has seizures with epileptic spasms and infantile hypotonia.

MRI brain shows nodular heterotopia.

He has tracheomalacia and mild subglottic narrowing and a ventricular and atrial septal defect. There is no evidence of structural renal abnormality. He has a left sided iris coloboma and visual impairment. He has had recurrent otitis media with effusions and mild to moderate bilateral hearing loss. He has a cleft palate.

He has eczema which is treated with topical steroids and emollients.

He has mild dysmorphic features including bitemporal narrowing, sparsity of the outer eyebrows, an upturned nose with a flat nasal bridge, a tented upper lip with downturned corners of his mouth. He has small toenails and transverse palmar creases.

**Individual 7: Maternally inherited p.Trp1100Cys**

This male individual is 4 years of age. There is no family history of neurodevelopmental disorders. He was born at term in good condition after a relatively uncomplicated pregnancy. A 2 vessel cord was identified on antenatal ultrasound. Birthweight was 3.288kg

He was delayed in all developmental milestones. At 4 years he has no babble and cannot pull to stand. He first sat at 2.5 years and bottom shuffled at 3 years of age. He had infantile hypotonia which has improved by the age of 4. He weight 14.2 kg has a height of 97.5 cm and head circumference of 49 cm at 4 years of age. He requires supplementary oral feeds and has persistent constipation that required treatment.

He had a small muscular ventricular septal defect which closed spontaneously and has had recurrent respiratory tract infections. He had developmental dysplasia of his hips, over-riding of his third toe by second and fourth toes and mild scoliosis. Distinctive facial features include brachycephaly, down-turned angles of the mouth, dysplastic ears with overfolded superior helices, full nasal tip and full lips

MRI shows delayed myelination, and undersulcation of right cortex with right perisylvian polymicrogyria, short corpus callosum, extra draining vein left side of cerebellum arising from torcula and terminating in sigmoid sinus, consistent with occipital sinus (normal variant)

The maternal X-chromosome inactivation pattern was markedly skewed (96:04) using the differentially methylated region within the ZMYM3 (ZNF261) gene.

**Individual 8: *De novo* p.Lys1549Arg**

This male individual is currently 2 years of age. He was delivered at 37 weeks after concerns with a non-reassuring fetal heart rate and a compound hand/vertex delivery. He was born in poor condition was an Apgar at 1 minute of 3 and 8 at 5 minutes. As he was limp, cyanotic and apnoeic he required positive pressure ventilation resuscitation. His birth weight was 2.65kg, head circumference 32.5 cm and length 51cm.

His weight is currently between the 10^th^ and 25th centile, height is less than the 1^st^ centile and weight is on the 75^th^ centile. He is 100% dependent on gastrostomy tube feeding and has severe dysphagia. He has abnormal epiglottis morphology. He has had recurrent aspiration and has excessive salivation.

He has a severe global developmental delay functioning at around a 3 month level at an age of 2 years. He smiles and cries, but does not engage with others. He is not yet sitting independently or starting to crawl. He cannot roll over. He is visually tracking. He has no verbal utterances and has never laughed. He can kick his legs and move his arms but is not yet reaching for objects. He cannot hold his head up when prone. He has not had clinical seizures but has some EEG abnormalities. He has a significant persistent global hypotonia.

He has had three MRI brain scans. On the first scan there was evidence of punctate intraparenchymal blood within the right parietal lobe and an additional area of blood within the extra-axial space of the posterior fossa. Myelination was judged to be appropriate for age. On repeat MRI there was evidence of mild generalised prominence of the extra-axial spaces with an interval increase in ventricular size. Corpus callosum was attenuated in the superior-interior dimension. There was a small cerebellum, particularly the inferior cerebellum. On the third scan there was further evidence of increased size of the ventricular system with prominence in particular of the third and fourth ventricle.

He has bilateral exotropia.

He has mild facial dysmorphism.

He has severe atopic eczema and seborrheic dermatitis.

**Individual 9: Maternally inherited Del-Exon37-38**

This male individual is currently 11 years of age. He was delivered by ventouse delivery at 40 weeks gestation and had initial difficulties sucking and swallowing and suboptimal weight gain. His birth weight was 3.2 kg, head circumference 33.3 cm and length 47cm.

His current head circumference is less than the 2^nd^ centile and weight less than the third centile. His height is between the 50^th^ and 75^th^ centile. He has persistent feeding difficulties with gastroesophageal reflux, and vomiting after meals and weight loss.

He has at least a moderate ID, with limited speech and delayed motor development. He has persistent central hypotonia and progressive appendicular spasticity. He appears to have a myopathy affecting his face, trunk and neck. Muscle biopsy showed pleomorphic mitochondria associated with an increase in lipid in some of the fibres suggestive but not diagnostic of fatty acid oxidation disorder. He has been investigated for possible absence seizures.

He has had recurrent lower respiratory tract infections due to aspirations and has laryngomalacia and mild cervical webbing.

He has had normal renal ultrasound scans but has cryptorchidism. He has intermittent esotropia. He has bilateral sensorineural hearing impairment (requiring cochlea implant) and recurrent otitis media with effusions.

He has scoliosis and bilateral talipes and flat acetabulae.

He has recurrently low neutrophils and basophils.

He has subtle dysmorphic features: including brachyplagiocephaly, a high palate, hypotelorism and mild bilateral blepharophimosis, underfolding of the ear helix, widely-spaced and cone shaped teeth. He has brachydactyly of his fingers and flat feet.

**Methods used for identifying the *THOC2* variants**

**Patient 1: *De novo* pArg77Cys**

Singleton whole genome sequencing (WGS) on the blood gDNA was performed at the Centre for Applied Genomics (Toronto, Canada) using established methods (Lionel et al., 2018). In brief, library preparation was performed from 500 ng of DNA using the Illumina TruSeq Nano DNA Library Preparation Kit (omitting the PCR amplification step) followed by sequencing on an Illumina HiSeq X platform as per Illumina’s recommended protocols. Base calling and data analysis were performed using Bcl2FASTQ or HiSeq Analysis Software (HAS) v2-2.5.55.1311 and reads were mapped to the hg19 reference sequence using BWA v0.7.12. SNVs and indels were detected using GATK (v3.4-46 or v3.7). Detected variants were annotated using a custom pipeline based on ANNOVAR as previously described (Lionel et al., 2018). The *THOC2* variant was confirmed by Sanger sequencing in a CAP/CLIA approved laboratory to be present in the male proband and absent in the mother (i.e., *de novo*). Mean coverage: 34.65X; % at >10X: 99%; % at >20X: 93%. No other candidate variants were identified.

**Individual 2: Maternally inherited p.Asn666Asp**

The exonic regions and flanking splice junctions were captured from the proband and parents’ gDNA using the IDT xGen Exome Research Panel v1.0. Massively parallel (NextGen) sequencing was performed on an Illumina system with 100bp or greater paired-end reads with a target coverage of >98.6% to a depth of at least 10×. Reads were aligned to human genome build GRCh37/UCSC hg19 and analysed for sequence variants using a custom-developed analysis tool. Additional sequencing technology and variant interpretation protocol has been described previously (Retterer et al., 2016). The general assertion criteria for variant classification are publicly available on the GeneDx ClinVar submission page (<http://www.ncbi.nlm.nih.gov/clinvar/submitters/26957/)>. A maternally-inherited variant in *PHYH:*c.135-2A>G, IVS2-2A>G was also identified, but no second variant in *PHYH* was identified and the patient did not have the clinical features of Refsum disease. Thus this variant was not considered relevant to the phenotype and the*THOC2* variant was the only variant in a gene compatible with the clinical presentation.

**Individual 3: *De novo* p.Lys724Glu**

Exome sequencing (ES) was performed on the blood gDNA. Details of the ES platform and filtering methods applied to identify putative variants are as reported (Deciphering Developmental Disorders, 2017). Three variants survived filtration: in addition to the *THOC2* variant there were two variants in the *APOE* gene. The *APOE* variants were not considered relevant to the phenotype. Thus, the *THOC2* variant was the only variant in a gene compatible with the clinical presentation.

**Individual 4: *De novo* p.Tyr881Cys**

Trio-based ES was performed using Agilent SureSelect CRE v2 kit on HiSeq4000 with 150-bp-paired-end reads. Reads were aligned to hg19 using BWA and variants were called using the GATK haplotype caller (reference: https://www.broad insti tute.org/gatk/). The *THOC2* variant was the only variant in a gene compatible with the clinical presentation.

**Individual 5a and 5b: Maternally inherited p. Cys981Tyr**

The gDNA was isolated from the patient’s and parents’ whole blood. Samples were prepared using the SeqCap EZ VCRome 2.0 (Roche NimbleGen) or the IDT xGen Exome Research Panel V1.0 (IDT). Quantified libraries were sequenced on Illumina flow cell using paired-end 100 or 150 cycle chemistry on the Illumina HiSeq or NextSeq. Initial data processing, base calling, alignments and variant calls were generated by various bioinformatics tools. Data were annotated with the Ambry Variant Analyzer tool (AVA). Sanger sequencing revealed the presence of this variant in the affected individual’s brother and mother and absent in his father. A karyotype and chromosomal microarray were reportedly normal. The *THOC2* variant was the only variant in a gene compatible with the clinical presentation.

**Individual 6: *De novo* p.Arg1075Tyr**

Trio ES was undertaken as previously described (Le Fevre et al., 2019). Briefly, the patient and his parents’ blood gDNAs were enriched for coding sequences using the Agilent SureSelect. All Exon v6 kit (total target size, 60 Mb;>23,000 genes). Paired-end reads were sequenced on an Illumina NextSeq 500 sequencer and the data was processed using the Exeter Genomics Laboratory in-house pipeline based on GATK (v3.4) best practice guidelines. Variants were filtered based on their frequency (variants with a MAF>0.001 were discarded) and genotype (e.g. our analysis discards inherited heterozygous variants). CNV analysis was included. We identified compound heterozygous variants in 3 genes (none known to cause human disease), maternally inherited X-linked variants in 4 genes (2 known disease genes)  and *de novo* heterozygous variants in 30 genes (4 known disease genes). The *THOC2* variant was the only variant in a gene compatible with the clinical presentation.

**Individual 7: Maternally inherited p.Trp1100Cys**

WGS was performed on the affected and parents recruited into the 100,000 Genomes Project (The National Genomics Research and Healthcare Knowledgebase v5, Genomics England. [doi:10.6084/m9.figshare.4530893.v5](https://doi.org/10.6084/m9.figshare.4530893.v5). 2019) (Turnbull et al., 2018). Reads were mapped to GRCh37 using the Isaac aligner and variants were called using the Isaac variant caller (Illumina). Structural variants were called with Manta and Canvas (Illumina). The samples were also genotyped using the InfiniumCoreExome-24v1 array (Illumina). A Tier 2 maternally-inherited variant (c.3300G>T p.Trp1100Cys) identified in the *THOC2* gene was confirmed by Sanger sequencing. The *THOC2* variant was the only variant in a gene compatible with the clinical presentation.

**Individual 8: *De novo* p.Lys1549Arg**

ES and variant identification was performed on the affected individual and his mother’s blood gDNA by GeneDx. Mother did not harbour the p.Lys1549Arg THOC2 variant. Briefly, the exonic regions and flanking splice junctions of the genome from gDNAs were captured and sequenced by massively parallel (NextGen) sequencing on an Illumina system with 100 bp or greater paired-end reads. Reads were aligned to human genome build GRCh37/UCSC hg19 and analyzed for sequence variants using a custom-developed analysis tool (Xome Analyzer). Clinically significant variants were confirmed by an appropriate orthogonal method in the proband and mother. Sequence and copy number alterations were reported according to the Human Genome Variation Society (HGVS) and International System for Human Cytogenetic Nomenclature (ISCN) guidelines, respectively. Mean depth of coverage was 99x with a quality threshold of 98.6%. No reportable secondary findings were identified in coding regions covered by the XomeDx test for the genes recommended to be analyzed and reported by the ACMG SF v2.0 (September, 2016). The identified p.Arg501Ter and p.Ser3247Ter FLG variants are most likely not relevant to the phenotype. Thus, the *THOC2* variant was the only variant in a gene compatible with the clinical presentation.

**Individual 9: Maternally inherited Del-Exon37-38**

Singleton ES was performed using Illumina TruSight One Expanded kit on Illumina NextSeq 550. Target coverage was >95% to a depth of at least 20x. Bioinformatics analysis filtering for rare (<1% VAF in population databases) coding and intronic ( +/- 10bp) variants in genes associated with RASopathies, Angelman-like disorders, and microcephaly, as well as copy number variant analysis for X-linked genes, identified the THOC2 microdeletion that would result in deletion of ex37-38 in the mRNA sequence. Further molecular studies showed its maternal inheritance. The *THOC2* variant was the only variant in a gene compatible with the clinical presentation.

**Supplementary Figure 1.** THOC2 variant amino acids are highly conserved. The amino acid sequences were aligned using [www.uniprot.org/align/](http://www.uniprot.org/align/) and the variant amino acids are shown in green.

**p.Arg77Cys p.Lys724Glu**

**Mutant** **DISEFCEDMPS 82 Mutant IRNTKESSQRL 729**

**Homo DISEFREDMPS 82 Homo IRNTKKSSQRL 729**

**Mus DISEFREDMPS 82 Mus IRNTKKSSQRL 729**

**Rattus DISEFREDMPS 82 Rattus IRNTKKSSQRL 729**

**Pan DISEFREDMPS 82 Pan IRNTKKSSQRL 729**

**Macaca DISEFREDMPS 82 Macaca IRNTKKSSQRL 729**

**Canis DISEFREDMPS 82 Canis IRNTKKSSQRL 729**

**Equus DISEFREDMPS 82 Equus IRNTKKSSQRL 729**

**Sus DISEFREDMPS 82 Sus IRNTKKSSQRL 729**

**Bos DISEFREDMPS 82 Bos IRNTKKSSQRL 729**

**Loxo DVSEFREDMPS 82 Loxo IRNTKKSSQRL 729**

**Gallus DIIEFREDMPS 79 Gallus IRNTKKSSQRL 726**

**Xenopus EIIAFRDDMPS 79 Xenopus IRNTKKSSQRL 725**

**p.Tyr881Cys p.Asn666Asp**

**Mutant ISPQFCATFWS 886 Mutant LQYVADQLKAG 671**

**Homo ISPQFYATFWS 886 Homo LQYVANQLKAG 671**

**Mus ISPQFYATFWS 886 Mus LQYVANQLKAG 671**

**Rattus ISPQFYATFWS 886 Rattus LQYVANQLKAG 671**

**Pan ISPQFYATFWS 886 Pan LQYVANQLKAG 671**

**Macaca ISPQFYATFWS 886 Macaca LQYVANQLKAG 671**

**Canis ISPQFYATFWS 886 Canis LQYVANQLKAG 671**

**Equus ISPQFYATFWS 886 Equus LQYVANQLKAG 671**

**Sus ISPQFYATFWS 886 Sus LQYVANQLKAG 671**

**Bos ISPQFYATFWS 886 Bos LQYVANQLKAG 671**

**Loxo ISPQFYATFWS 886 Loxo LQYVANQLKAG 671**

**Gallus ISPQFYATFWS 883 Gallus LQYVANQLKAG 668**

**Xenopus ISPQFYATFWS 882 Xenopus LQYVANQLKAG 667**

**p.Cys981Tyr p.Arg1075Trp**

**Mutant KFLQLYIFPRC 986 Mutant FLTILWATGFD 1080**

**Homo KFLQLCIFPRC 986 Homo FLTILRATGFD 1080**

**Mus KFLQLCIFPRC**  **986 Mus FLTILRATGFD 1080**

**Rattus KFLQLCIFPRC 986 Rattus FLTILRATGFD 1080**

**Pan KFLQLCIFPRC**  **986 Pan FLTILRATGFD 1080**

**Macaca KFLQLCIFPRC 986 Macaca FLTILRATGFD 1080**

**Canis KFLQLCIFPRC 986 Canis FLTILRATGFD 1080**

**Equus KFLQLCIFPRC 986 Equus FLTILRATGFD 1080**

**Sus KFLQLCIFPRC 986 Sus FLTILRATGFD 1080**

**Bos KFLQLCIFPRC 986 Bos FLTILRATGFD 1080**

**Loxo KFLQLCIFPRC 986 Loxo FLTILRATGFD 1080**

**Gallus KFLQLCIFPRC 983 Gallus FLTILRATGFD 1077**

**Xenopus KFLQLCIFPRC 982 Xenopus FVTILRAASFD 1076**

**p.Trp1100Cys p.Lys1549Arg**

**Mutant H**VVHKCHYKLT 1105 Mutant SFKSERMDKIS 1554

Homo HVVHKWHYKLT **1105 Homo SFKSEXMDKIS 1554**

**Mus HVVHKWHYKLT 1105 Mus SFKSEKMDKIS 1554**

**Rattus HVVHKWHYKLT 1105 Rattus SFKSEKMDKIS 1554**

**Pan HVVHKWHYKLT 1105 Pan SFKSEKMDKIS 1554**

**Macaca HVVHKWHYKLT 1105 Macaca SFKSEKMDKIS 1554**

**Canis HVVHKWHYKLT 1105 Canis SFKSEKMDKIS 1554**

**Equus HVVHKWHYKLT 1105 Equus SFKSEKMDKIS 1554**

**Sus HVVHKWHYKLT 1105 Sus SFKSEKMDKIS 1554**

**Bos HVVHKWHYKLT 1105 Bos SFKSEKMDKIS 1554**

**Loxo HVVHKWHYKLT 1105 Loxo SFKSEKMDKIS 1554**

**Gallus HVVHKWHYKLT 1102 Gallus SIKAEKMEKSS 1555**

**Xenopus HVVHKWHYKLT 1101 Xenopus FMKSEKMEKSS 1551**

**REFERENCES**

Deciphering Developmental Disorders, S. (2017). Prevalence and architecture of de novo mutations in developmental disorders. *Nature* 542**,** 433-438.

Le Fevre, A., Baptista, J., Ellard, S., Overton, T., Oliver, A., Gradhand, E., et al. (2019). Compound heterozygous Pkd1l1 variants in a family with two fetuses affected by heterotaxy and complex Chd. *Eur J Med Genet***,** 103657.

Lionel, A.C., Costain, G., Monfared, N., Walker, S., Reuter, M.S., Hosseini, S.M., et al. (2018). Improved diagnostic yield compared with targeted gene sequencing panels suggests a role for whole-genome sequencing as a first-tier genetic test. *Genet Med* 20**,** 435-443.

Retterer, K., Juusola, J., Cho, M.T., Vitazka, P., Millan, F., Gibellini, F., et al. (2016). Clinical application of whole-exome sequencing across clinical indications. *Genet Med* 18**,** 696-704.

Tellegen P. J., W., M., Wijnberg‐Williams, B. J., Laros, J. A. (1998). *Snijders‐Oomen Nonverbal Intelligence Test. SON‐R 2.5–7. Manual and research report.* Lisse, the Netherlands.

Thompson, H.L., Viskochil, D.H., Stevenson, D.A., and Chapman, K.L. (2010). Speech-language characteristics of children with neurofibromatosis type 1. *Am J Med Genet A* 152A**,** 284-290.

Turnbull, C., Scott, R.H., Thomas, E., Jones, L., Murugaesu, N., Pretty, F.B., et al. (2018). The 100 000 Genomes Project: bringing whole genome sequencing to the NHS. *BMJ* 361**,** k1687.
